# Transforming undergraduate education in geriatric medicine: an innovative curriculum at Bristol Medical School

**DOI:** 10.1007/s41999-022-00690-w

**Published:** 2022-09-07

**Authors:** Grace M. E. Pearson, Tomas Welsh, Lucy V. Pocock, Yoav Ben-Shlomo, Emily J. Henderson

**Affiliations:** 1 Bristol Medical School (Population Health Sciences), University of Bristol, Bristol, UK; 2 Royal United Hospitals NHS Foundation Trust, Bath, UK

**Keywords:** Undergraduate, Geriatric medicine, Medical education, Teaching innovation, Complexity

## Abstract

The World Health Organization (WHO) advocates investment in high-quality undergraduate education in geriatric medicine as a means of meeting the future needs of the aging population. However, there is a lack of evidence for the optimal delivery of training in this area. Rigorous pedagogical research is required to determine the most effective way to equip tomorrow’s doctors with the skills and knowledge to care for older adults with complex health and social care needs. The transition between two undergraduate medical curricula meant that Bristol Medical School (BMS) was uniquely positioned to innovate and evaluate undergraduate education in geriatric medicine. This transition marked BMS’ departure from a ‘traditional’ curriculum to case-based learning. The outgoing curriculum included a 4-week unit in geriatrics, whilst the new programme includes an 18-week clerkship titled ‘Complex Medicine in Older People’ (CMOP). CMOP is a clinical clerkship with 18 cases at its core, covering the fundamental aspects of geriatric medicine. The core cases and clinical learning are enhanced with five expert lectures, six tutorials and three journal clubs. Reflective practice is modelled and promoted with Balint groups and a book club. Consolidative workplace-based assessments and clinical portfolio mirror those used in postgraduate training, preparing students for professional practice. CMOP is iteratively improved in real-time using staff and student feedback. This marked shift in mode and duration of teaching affords the opportunity to evaluate the impact of differing education in geriatrics, providing an evidence-based model for teaching on aging.

**Supplementary Information:**

The online version contains supplementary material available at 10.1007/s41999-022-00690-w.

## Key summary points

**Aim:**

Research and innovation in undergraduate education in geriatric medicine is essential to effectively equip tomorrow’s doctors with the skills and knowledge required to care for older adults with complex health and social care needs.

**Findings:**

Transitioning between two undergraduate medical curricula meant that Bristol Medical School was uniquely positioned to innovate and evaluate undergraduate education in geriatric medicine. The product of this was a new innovative 18-week programme titled ‘Complex Medicine in Older People’, outlined in this article.

**Message:**

This marked shift in mode and duration of teaching affords the opportunity to innovate and evaluate undergraduate education in geriatrics, providing an evidence-based model for future teaching on aging.

**Supplementary Information:**

The online version contains supplementary material available at 10.1007/s41999-022-00690-w.

## Background

Investment in the education and training of doctors is a means of anticipating the challenges of the aging population. Recognising medical students as ‘agents of change’ [1], the World Health Organisation (WHO) undertook international studies of undergraduate education in geriatric medicine, finding that the quality of education in geriatrics at medical school positively influenced medical students’ attitudes towards older people, making them more likely to consider a career as a specialist geriatrician [2]. However, 27% of participating medical schools offered no teaching on geriatrics [3], and in the UK, 15.8% of medical students received no teaching in the specialty, highlighting inadequacies in UK medical training that needed to be addressed urgently [2]. Hence, investment in undergraduate education in geriatrics has wide-reaching potential to (a) positively impact the knowledge and skills of ‘tomorrow’s doctors’, (b) tackle systemic ageism by improving attitudes amongst healthcare professionals and (c) reduce workforce pressures by producing more clinicians specialising in geriatrics.

Bristol Medical School (BMS), part of the University of Bristol, is a leading UK institution for undergraduate medical training, located in the Southwest of England. In 2017, BMS began a radical curriculum redesign, marking its departure from a ‘traditional’ pre-clinical/clinical curriculum into ‘case-based learning’, with clinical attachments, termed ‘clerkships’, as early as year 1. The outgoing curriculum included a 4-week unit in geriatrics, which was a typical reflection of how geriatric medicine had been taught across the UK to date [4]. The new programme (MB21) involves an 18-week clerkship in the 4th year of study called ‘Complex Medicine in Older People’ (CMOP).

This marked curriculum transformation has afforded an opportunity to both innovate and evaluate undergraduate education in geriatric medicine, which will provide one model for teaching on aging, complementing the work of the European Geriatric Medicine Society [5]. We anticipate that by providing this high-quality education in geriatric medicine, we will equip and inspire physicians of the future with the expertise, knowledge and skills needed to care for the unique and complex needs of older people.

## Complex Medicine in Older People curriculum

**Fig. 1:**
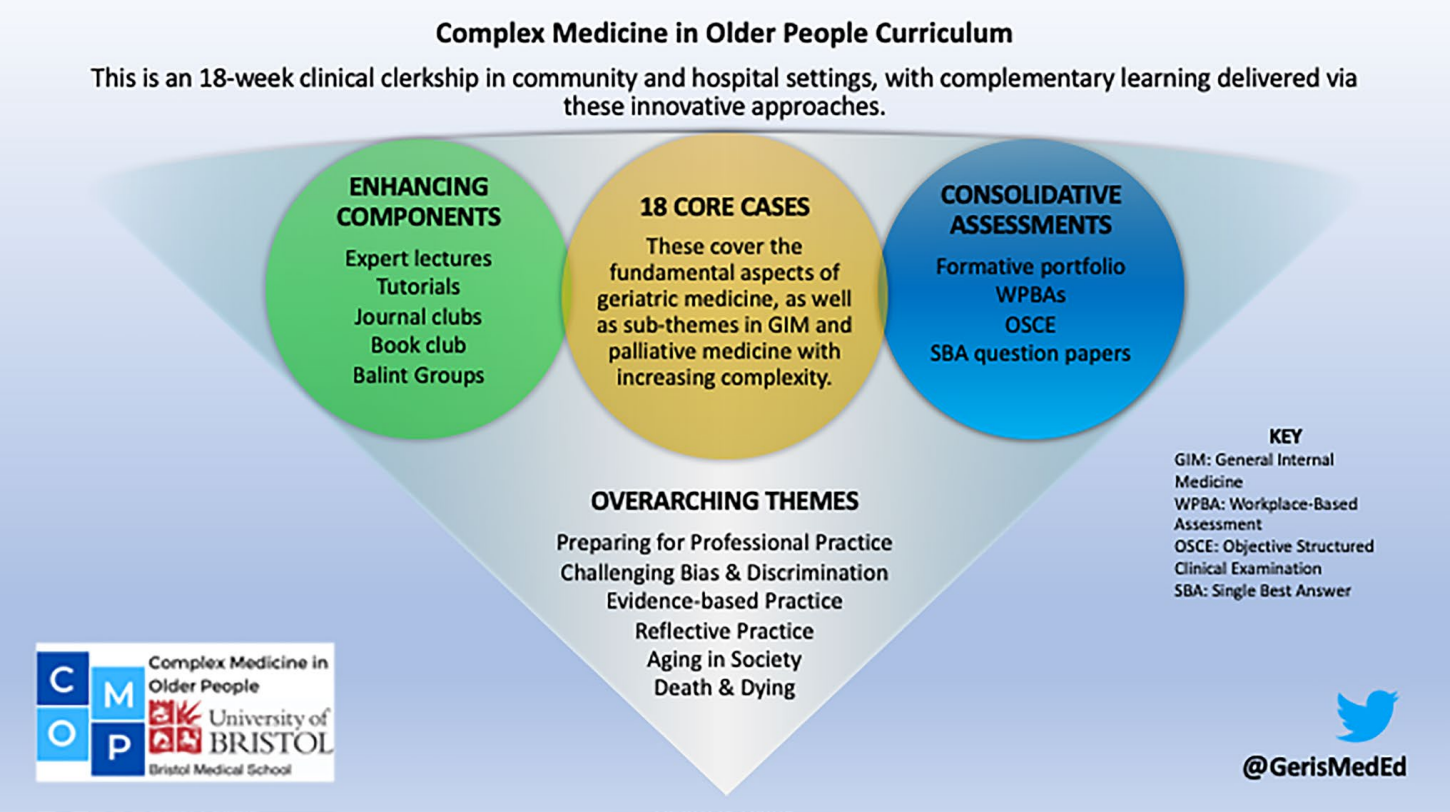
An overview of the CMOP curriculum, which has 18 cases at its core, with other innovative components designed to enhance and consolidate case-based learning and clinical attachments in geriatric medicine, general internal medicine, and palliative medicine

CMOP was designed to ensure that students experienced the breadth and complexity of geriatrics, incorporating perspectives from both science and humanities, as well as challenging preconceptions of aging and dying, as outlined in Fig. 1. CMOP is, however, first-and-foremost a clinical clerkship which students undertake at seven National Health Service (NHS) hospitals across the Southwest, known as ‘clinical academies’. This takes up half of the teaching time in year 4, meaning that two cohorts of approximately 100 students undertake the clerkship each year. The core of CMOP is eighteen cases, that have been developed in-line with existing recommended undergraduate curricula (Table 1) [6, 7]. These cases are designed to give a comprehensive coverage of geriatric medicine, and sub-themes of general internal medicine and palliative medicine. The case scenarios are specifically designed to be “real world” and become increasingly complex as the weeks progress to challenge and stimulate the students to think more broadly and holistically beyond specific pathologies. Further innovations are embedded within the programme to enhance and consolidate case-based learning and clinical attachments in geriatrics.

**Table 1:** An overview of the topics covered in weekly case-based learning

Week	Case title	Week	Case title
1	Comprehensive assessment of the older person	10	Dementia
2	Frailty in acute illness	11	Delirium
3	Frailty vs Multimorbidity	12	Parkinson’s Disease
4	Falls	13	Peri-operative care of older people
5	Polypharmacy	14	Skin & Nutrition
6	Stroke & Dysphagia	15	Mental health in older people
7	Collapse & Dizziness	16	Recognition of dying
8	Fragility fractures	17	Integrated care for older people
9	Continence & Heart Failure	18	Final round-up

### ‘Hub’ lectures

Five ‘Hub’ lectures are delivered by BMS subject experts on specialist topics: the physiology of aging, end-of-life care, health economics, evidence-based practice, and medical ethics. These were chosen as expertise in the topics is based centrally at the University and delivering them locally would be problematic, with large variability in the teaching experience. The Hubs last approximately 90-min, and are broadcast online to all the clinical academies at regular intervals, bringing all the students together into a common learning space. Interactivity and teamwork are encouraged through use of online education tools, such as ‘Mentimeter’ (www.mentimeter.com), that enliven debate and consolidate learning with instant feedback on questions posed [8].

### Tutorials

Six practical tutorials are delivered in small groups within the clinical academies, varying in length and content. The tutorials are ordered to mimic an older patient’s journey through an admission to hospital, beginning with a session on ‘how to clerk an older person’, highlighting the adaptations to history-taking and examination required to capture the complexity of unwell older people. This is followed by simulation teaching on ‘how to ward round’ [9]. Both of these tutorials were developed in response to need and reflect the cornerstones of inpatient medical practice. Subsequent sessions explore ‘what it’s like to grow old’ where students are asked to perform everyday tasks while dressed in aging suits which mimic the physical impairments encountered with aging [10]. This is complemented by a communication skills tutorial where students learn how to adapt their consultation style to disabilities commonly encountered in caring for older people, such as altered vision, hearing loss and cognitive impairment. In order to prepare students for professional practice, CMOP concludes with two tutorials on ‘what to do when a patient dies’ and ‘what to do when someone makes a complaint’, which are delivered by non-clinical staff from the bereavement and patient liaison services at the academies. 

### Journal clubs and ‘meet the author’ interviews

Appraisal of evidence and translation of this into practice is fostered through three journal clubs and research papers embedded within the eighteen cases. These sessions allow them to put into practice and further consolidate past teaching in Evidence Based Practice and prepare the students for postgraduate experience. Short online video interviews with academics who have published in the fields have been interwoven throughout the curriculum to make medical literature more accessible to undergraduates and connect students with the rich network of scholars researching aging locally.

### Book club

It is widely accepted that caring for older people requires a holistic approach that cannot be learnt exclusively from medical textbooks. To highlight the human aspects of geriatric medicine, the curriculum includes a shortlist of eleven popular books that explore issues around aging and dying in an engaging and accessible manner (listed in the Supplementary Appendix). Students meet in groups twice to discuss and reflect on their reading, and afterwards are encouraged to continue the dialogue and engage with the wider world of geriatrics on Twitter using the hashtag #CMOPBookClub.

### Balint groups

Acknowledging that certain aspects of aging and dying might be emotionally challenging, we provide a space where students can talk freely about being involved in the care of older people. Students participate one hour-long Balint groups on four occasions where reflective practice is modelled and promoted, with a particular focus on student-patient interactions rather than clinical content (www.balint.co.uk) [11]. These groups are facilitated by early-career, specialist trainees in geriatric medicine, general practice, and psychiatry, minimising any perceived hierarchy and promoting peer-to-peer mentorship.

### Tackling ageism

**Fig. 2:**
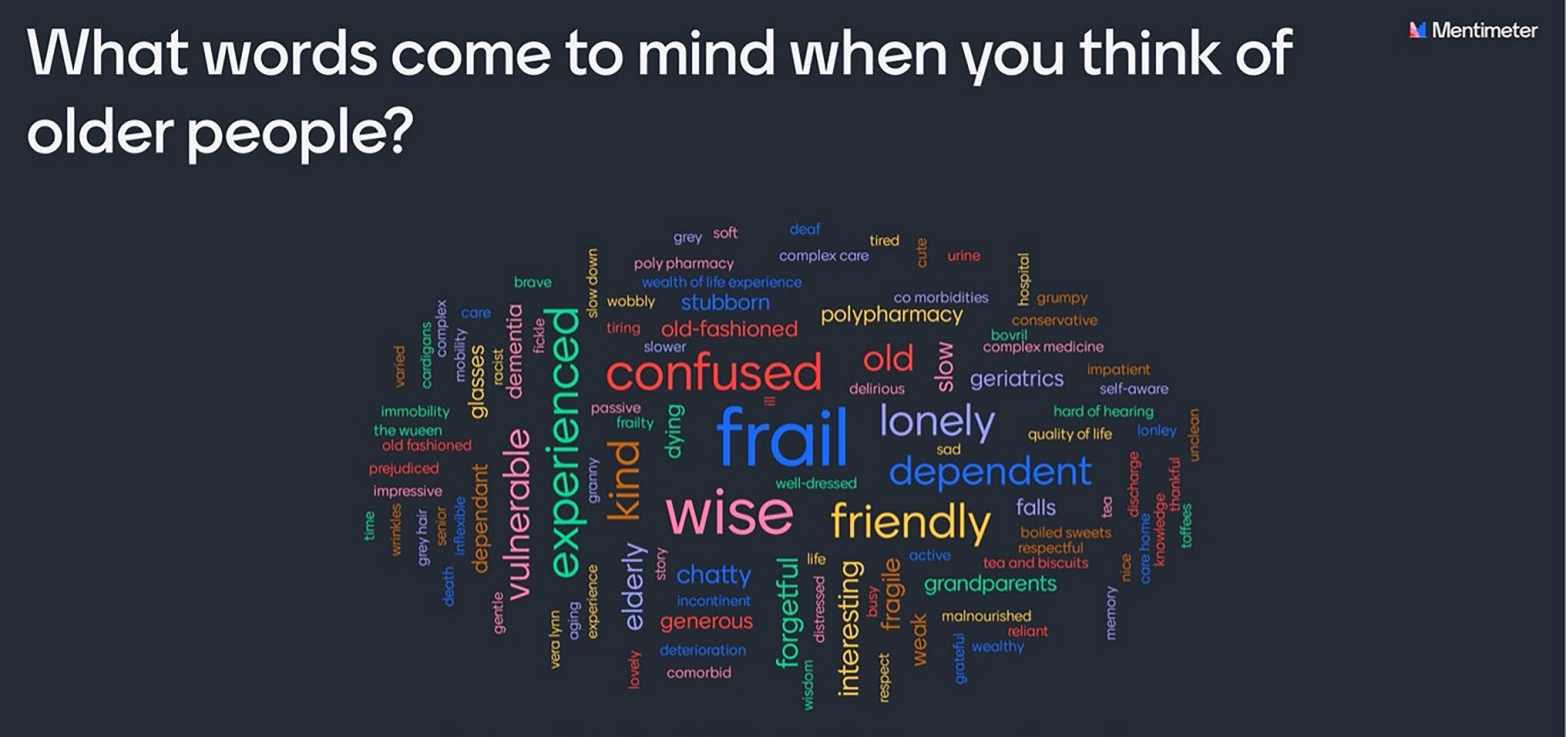
An example word cloud generated on Mentimeter during Hub 1 by students asked what words they associate with ‘older people’ [8]

Ageism is a universal problem that has detrimental effects on the healthcare of older people worldwide [12, 13] and challenging ageism from the earliest possible stage is essential to prevent the development of negative attitudes amongst clinicians. In CMOP, students reflect on the portrayal of older people in television advertising (Hub 1) and popular literature (Book Club). In their case-based learning and clinical practice, students reflect on commonly encountered pejorative terms, such as ‘elderly’, ‘acopia’, ‘pleasantly confused’ and ‘failed discharge’﻿ [14]. They are tasked to reflect on their own unconscious biases towards older people and how this might impact their clinical practice—one example of this being the word clouds and subsequent discussions generated by students when asked which words they associated with ‘older people’ (Fig. 2).

### Formal assessments and continuous improvement

Formative assessment in CMOP takes the form of a portfolio which guides and documents the students’ clinical activities. Early data reveals that students appreciate the structure that the portfolio brings to this long clerkship. They are required to complete workplace-based assessments (case-based discussions and mini-clinical evaluation exercises), which mimic those used in postgraduate medical training in the UK—again, preparing the students for professional practice [15]. Summative assessment takes place at the end of year 4 in an Objective Structured Clinical Examination (OSCE) and single-best answer question papers. Feedback, collected in real-time from students and staff, is used to iteratively improve the curriculum and learning materials.

## Lessons learnt

A ‘modular’ curriculum design was chosen to complement the core case-based learning. This non-prescriptive approach made it easier logistically for academies to adapt and implement the curriculum to suit their students and healthcare services, particularly during the upheaval of COVID-19. We anticipate that other institutions might adopt some of these modular components of the curriculum to segue with their existing frameworks and learning opportunities.

We have found that student attendance can diminish in the latter stages of the 18-week clerkship and attrition is likely due to the approach of examination season at the end of year 4. We have tackled this by expanding the mandatory portfolio requirements between iteration 1 (2020/21) and 2 (2021/22), and by adding more general medicine ‘revision’ within case-based learning. We also piloted a tutorial exploring professionalism, particularly related to attendance, which the students rated positively but were clear that they would have found it more useful in earlier years.

Our primary challenge was at inception, when arguing for the increased duration of CMOP within the new MB21 programme; Other specialities did not immediately recognise the value of an expanded geriatrics curriculum and in some cases felt that this detracted from time spent in their specialty. We argued that curriculum time should reflect the demographic of diseases and population that new graduates will serve. In addition, the health and social challenges of multi-morbidity are embedded here within geriatric teaching, but are applicable to many more specialties. A rigorous and embedded programme of ongoing pedagogical research supports our case, with longer-term outcomes awaited. This serves to continually iterate and improve the teaching and curriculum.

## Supplementary Information

Below is the link to the electronic supplementary material.

Supplementary file1 (DOCX 14 KB)
