# Supplementary material for: Transforming undergraduate education in geriatric medicine: an innovative curriculum at Bristol Medical School
Source: Eur Geriatr Med. 2022 Sep 7;13(6):1487–91. doi: 10.1007/s41999-022-00690-w (PMC9451112; doi:10.1007/s41999-022-00690-w)
Supplement: Supplementary file 1 — Supplementary file1 (DOCX 14 KB) [file 41999_2022_690_MOESM1_ESM.docx]

**SUPPLEMENTARY APPENDIX**

**CMOP BOOK CLUB READING LIST**

Fiction:

- Thursday Murder Club (Richard Osman)
- Elizabeth is Missing (Emma Healey)
- The 100-year-old man Who Climbed Out of the Window and Disappeared (Jonas Jonasson)
- The Little Old Lady Who Broke All the Rules (Catharina Ingelman-Sundberg)
- The Carer (Deborah Moggach)
- Allelujah! (Alan Bennett)

Non-Fiction:

- The Book About Getting Older (Lucy Pollock)
- With the End in Mind (Kathryn Mannix)
- Being Mortal (Atul Gawande)
- Somewhere Towards the End (Diana Athill)
- Somebody I Used to Know (Wendy Mitchell)
